# Prospective benchmarking of an observational analysis in the SWEDEHEART registry against the REDUCE-AMI randomized trial

**DOI:** 10.1007/s10654-024-01119-3

**Published:** 2024-05-08

**Authors:** Anthony A. Matthews, Issa J. Dahebreh, Conor J. MacDonald, Bertil Lindahl, Robin Hofmann, David Erlinge, Troels Yndigegn, Anita Berglund, Tomas Jernberg, Miguel A. Hernán

**Affiliations:** 1 Unit of Epidemiology, Institute of Environmental Medicine, Karolinska Institutet, Nobels Väg 13, 171 65 Solna, Stockholm, Sweden; 2 CAUSALab, Department of Epidemiology, Harvard T.H. Chan School of Public Health, Boston, USA; 3 Department of Biostatistics, Harvard T.H. Chan School of Public Health, Boston, USA; 4 Department of Medical Sciences, Cardiology, Uppsala University, Uppsala, Sweden; 5 Uppsala Clinical Research Center, Uppsala, Sweden; 6 Division of Cardiology, Department of Clinical Science and Education, Karolinska Institutet, Södersjukhuset, Stockholm, Sweden; 7 Department of Cardiology, Clinical Sciences, Skåne University Hospital, Lund University, Lund, Sweden; 8 Department of Clinical Sciences, Danderyd University Hospital, Karolinska Institutet, Stockholm, Sweden

**Keywords:** Benchmarking, Target trials, Randomized trials, Causal inference

## Abstract

Prospective benchmarking of an observational analysis against a randomized trial increases confidence in the benchmarking process as it relies exclusively on aligning the protocol of the trial and the observational analysis, while the trials findings are unavailable. The Randomized Evaluation of Decreased Usage of Betablockers After Myocardial Infarction (REDUCE-AMI, ClinicalTrials.gov ID: NCT03278509) trial started recruitment in September 2017 and results are expected in 2024. REDUCE-AMI aimed to estimate the effect of long-term use of beta blockers on the risk of death and myocardial following a myocardial infarction with preserved left ventricular systolic ejection fraction. We specified the protocol of a target trial as similar as possible to that of REDUCE-AMI, then emulated the target trial using observational data from Swedish healthcare registries. Had everyone followed the treatment strategy as specified in the target trial protocol, the observational analysis estimated a reduction in the 5-year risk of death or myocardial infarction of 0.8 percentage points for beta blockers compared with no beta blockers; effects ranging from an absolute reduction of 4.5 percentage points to an increase of 2.8 percentage points in the risk of death or myocardial infarction were compatible with our data under conventional statistical criteria. Once results of REDUCE-AMI are published, we will compare the results of our observational analysis against those from the trial. If this prospective benchmarking is successful, it supports the credibility of additional analyses using these observational data, which can rapidly deliver answers to questions that could not be answered by the initial trial. If benchmarking proves unsuccessful, we will conduct a “postmortem” analysis to identify the reasons for the discrepancy. Prospective benchmarking shifts the investigator focus away from an endeavour to use observational data to obtain similar results as a completed randomized trial, to a systematic attempt to align the design and analysis of the trial and the observational analysis.

**Supplementary Information:**

The online version contains supplementary material available at 10.1007/s10654-024-01119-3.

## Background

An analysis of observational data can complement a randomized trial by addressing questions that could not be answered by the trial. For example, observational analyses may sometimes be used to extend inferences made in the trial to a longer follow-up, or to populations not included or underrepresented in the trial. Attempts at extending inferences from a trial can be supported by benchmarking, that is, a systematic comparison of an observational analysis and a trial that aim to ask the same question [1–3]. To increase confidence in the benchmarking process, it is preferable to complete the observational analysis before the trial findings are known. Investigators can then be confident that data have not been manipulated until they produce estimates that agree with the trial. Also, if possible, the observational analysis would be undertaken in the same population from which trial participants were recruited [4–7].

As an example, the Randomized Evaluation of Decreased Usage of Betablockers After Myocardial Infarction (REDUCE-AMI) randomized trial [8], mainly embedded in the Swedish Web-system for Enhancement and Development of Evidence-based care in Heart disease Evaluated According to Recommended Therapies (SWEDEHEART) registry in Sweden [9], started recruitment in September 2017 and is expected to report initial results in 2024. Because the SWEDEHEART registry also contains data on individuals with myocardial infarction before September 2017, there is an opportunity to design an observational analysis that emulates a trial similar to REDUCE-AMI using data from the same underlying population.

Benchmarking such an observational analysis against the, currently unknown, results of REDUCE-AMI will be deemed “successful” if the same clinical decision would be made on the basis of either source of evidence (i.e., using the trial or the observational analysis results). If this prospective benchmarking is successful, it supports the credibility of analyses using the same observational data to rapidly provide answers to questions that could not be answered by the initial trial. If benchmarking is not successful, we will conduct a “postmortem” analysis to identify the reasons for the discrepancy, which will inform future observational analyses.

Here, we design a target trial as similar as possible to the REDUCE-AMI randomized trial, and then emulate the target trial using observational data from the SWEDEHEART and linked registers.

## The index trial: REDUCE-AMI

REDUCE-AMI (ClinicalTrials.gov ID: NCT03278509) is an ongoing registry-based, prospective, randomized, open-label, parallel trial that started recruitment on 11th September 2017 in Sweden (38 centers), Estonia (1 center), and New Zealand (6 centers). An overview of the trial’s protocol is in Table 1. Briefly, individuals are deemed eligible within seven days of type 1 myocardial infarction with preserved left ventricular systolic ejection fraction with a coronary angiography that documented obstructive coronary artery disease. Major exclusion criteria are any indication (other than for secondary prevention) or contraindication for beta blocker treatment according to the treating physician, and any condition that may affect their ability to comply with the study protocol.

**Table 1:** Outline of protocols of the REDUCE-AMI trial, a similar target trial, and of an emulation of the target trial using the SWEDEHEART Registry

Protocol component	Index trial: REDUCE-AMI	Target trial	Target trial emulation using SWEDEHEART^a^
Eligibility criteria	• Age ≥ 18 years from 11th Sept 2017 to time when 379 primary endpoints have occurred • Day 1–7 of hospitalization for type 1 myocardial infarction in participating hospitals in Sweden, Estonia, and New Zealand • Obstructive coronary artery disease documented by coronary angiography • Normal ejection fraction (EF ≥ 50%) confirmed by post-MI echocardiography • No contraindications for beta blockers • No beta blocker indications other than as secondary prevention according to treating physician • No conditions that may influence the patient’s ability to comply with study protocol	Same as index trial apart from: • 1st Sept 2010 to 10th Sept 2017 in Sweden only •Day 1–30 after hospitalization for type 1 myocardial infarction on which coronary angiography is performed • “Contraindications for beta blockers” operationalized as bradycardia, Av-block II-III, hypotension, syncope, asthma, COPD, or stroke in previous 3 years • “No beta blocker indication” operationalized as no beta blocker use in previous 3 years, heart rate ≥ 120, systolic blood pressure ≥ 180, diastolic blood pressure ≥ 120, history of chronic heart failure, or atrial flicker/flutter • “Conditions that may influence ability to comply” operationalized as psychiatric disorders or dementia in previous 3 years • On statins and antithrombotics at baseline	Same as target trial • All diagnoses (apart from beta blocker indications and dementia) identified as primary or secondary diagnosis in either the inpatient or outpatient sub registers of the Patient Register within three years of baseline • Beta blocker indications and dementia and identified using SWEDEHEART registry • Beta blocker use identified from the prescribed drug register in prior 3 years or a record of beta blocker use on admission to hospital from SWEDEHEART registry • Statins and antithrombotics identified from the prescribed drug register
Treatment strategies	(1) Beta blockers—Long-term oral beta blockers (metoprolol succinate or bisoprolol) unless a contraindication arises. The treating physician encouraged to aim for a dose of ≥ 100 mg for metoprolol succinate and ≥ 5 mg for bisoprolol (2) No beta blockers unless indicated for reasons other than secondary prevention following myocardial infarction—Discouraged to use beta blockers as long as there is no other indication than strictly secondary prevention after myocardial infarction	Same as index trial • Contraindications to beta blockers operationalized as above • Indications for beta blockers operationalized as hypertension, angina, arrhythmia, heart failure	Same as target trial apart from: • Prescription dates from Prescribed Drug register. If no record, but SWEDEHEART indicates beta blocker at discharge, prescription date set at discharge • Contra- and indications, primary or secondary diagnosis in inpatient or outpatient register • Metoprolol and bisoprolol assumed to be daily dose of 100 mg and 5 mg respectively • Date of non-adherence based on number of pills and pill dose, divided by daily dose (continuous if gap < 180d)
Treatment assignment	Individuals are randomly assigned to a treatment strategy and are aware of their assignment	Same as index trial	• Individuals assigned to strategy data compatible with at baseline and assignment to beta blockers operationalized as a prescription. Assignment assumed random within levels of baseline covariates in Supplementary Table 1
Outcomes	*Primary*: Death or myocardial infarction identified from the Swedish Total Population register and SWEDEHEART respectively *Secondary*: Each component of primary outcome	Same as index trial	Same as target trial
Follow-up	Starts at assignment, ends at first outcome, migration, or end of follow up (yet to be defined)	Same as index trial apart from: • End of follow at the earliest of 31st December 2017 or five years after start of follow up	Same as target trial apart from: • Unable to identify migration date
Contrasts	Intention to treat effect	Intention-to-treat and per protocol effects	Same as target trial
Statistical analysis	• Absolute risks estimated via Kaplan Meier • Hazard ratios estimated using Cox regression	Same as index trial apart from: Absolute risks estimated using pooled logistic regression model with adjustment for unbalanced baseline covariates via IP weighting. Hazard ratio estimated from same model without product terms with time Per protocol analysis similar except individuals censored when they deviate from assigned treatment, and IP weighting to adjust for baseline and time-varying variables	Same as target trial. Baseline covariates listed in Supplementary Table 1

^a^All ICD-10 and ATC codes used to operationalize diagnoses and treatments in Supplementary Table 4

Consenting individuals are randomly assigned to either beta blockers or no beta blockers. Individuals randomized to beta blockers are administered the assigned treatment (metoprolol or bisoprolol) during the hospital stay and receive a prescription for continued use after discharge. The treating physician is encouraged to aim for a daily dose of at least 100 mg for metoprolol or 5 mg for bisoprolol, and participants are encouraged to continue the beta blockers indefinitely following discharge, unless contraindications develop. Individuals randomized to no beta blockers are discouraged from using beta blockers as long as there is no new indication. All individuals recruited into the trial receive written information about the importance of continuing the assigned treatment and an ID-card size with the same information in case of medical contact. For blood pressure control, guidelines recommend treatments other than beta blockers as first-line treatment. If an individual is already using beta blockers when enrolled into the study and assigned to no treatment, a tapering of the beta blocker is carried out during the following two to four weeks. It is recommended that tapering ends with at least 4 days of lowest possible dose, corresponding to 12.5 mg metoprolol or 1.25 mg bisoprolol.

The primary outcome is the composite of death from any cause or new myocardial infarction. Information on death is obtained from the Swedish Total Population Register; information on new myocardial infarction during the initial hospital stay and readmission due to a non-fatal myocardial infarction is collected from the SWEDEHEART registry. To estimate the intention-to-treat effect, all individuals will be included in an intention-to-treat analysis, which will be based on events of all follow-up time of each individual from randomization to end of follow-up. Absolute risks of all endpoints will be estimated using Kaplan–Meier curves in each group. Hazard ratios and their 95% confidence interval will be estimated via Cox proportional hazards regression. The trial stopped recruitment on 3rd May 2023, at which point 5014 individuals had been enrolled [8].

## The observational analysis

Causal inference from observational data can be seen as an attempt to emulate a pragmatic randomized trial—the target trial—that would answer the question of interest. The approach for emulating a target trial has 2 steps: 1) specify the protocol of the target trial, and 2) emulate the target trial using observational data and appropriate methods [10–13]. The target trial framework helps articulate a precise causal question, assists with discussions about trade-offs regarding study design, and facilitates comparisons with randomized trials that aim to ask the same question as the observational study. Explicit target trial emulation means the only unavoidable difference between the target trial and its emulation is how treatment is assigned to eligible individuals at the start of follow-up: at random in the target trial; under clinical practice in the emulation. A successful emulation that relies on conditional exchangeability at baseline, therefore, requires detailed data on baseline confounders.

### The target trial

To compare REDUCE-AMI to an observational analysis that attempts to answer similar clinical questions, we first specified the protocol of a target trial similar to the protocol of REDUCE-AMI, with deviations only when the observational data did not correspond to the information collected in the trial (see also Table 1) [10]. Here, we briefly discuss the main differences.

Recruitment for the target trial would only be in the Swedish centers participating in REDUCE-AMI (not Estonia or New Zealand) and would run from 1st September 2010 until 10th September 2017 (the day before the start of recruitment for REDUCE-AMI); this difference only reflects data availability. The eligibility criteria are the same as for the index trial except that eligibility extends through 30 days after angiography, individuals are required to have received statin and anti-thrombotic treatment (because most individuals who initiate beta blockers also receive these two treatments), and no prior use of beta blockers is allowed (because we cannot reliably emulate a protocol-mandated tapering of beta blockers in the no beta blocker group). Table 1 describes the operationalization of contraindications and indications to beta blockers, as well as of conditions that limit an individual’s ability to adhere to the assigned treatment. The treatment strategies and outcomes are the same as REDUCE-AMI, and follow-up ends at the earliest of 31st December 2017, five years after baseline, or at the outcome of interest. The causal contrasts in the target trial are the intention-to-treat and per-protocol effects.

The intention-to-treat analysis is the same as for the index trial under the assumption that assignment to beta blockers in the index trial is analogous to the (more pragmatic) target trial where assignment would be a beta blocker prescription. Absolute risks can be estimated nonparametrically via Kaplan–Meier or parametrically, using a smooth function of time, by fitting a pooled logistic regression model with an indicator for assigned strategy, time of follow-up (restricted cubic spline with knots at 6, 12, 24, and 48 months), and a product term between the assignment indicator and time of follow-up. The 5-year risk in each group is then compared via risk differences and ratios. An estimate analogous to the hazard ratios from a Cox regression model can be obtained using the pooled logistic regression model without the assignment—time product term [14]. Inverse probability (IP) weighting can be used to adjust for prognostic factors that are imbalanced at baseline, that is, each individual receives an IP weight whose denominator is, informally, the probability of being assigned to the individual’s assigned group conditional on the prognostic factors. These probabilities can be estimated via a logistic regression model [15]. To estimate the total effect on myocardial infarction, individuals who die are treated as not experiencing the outcome after death [16].

The per protocol analysis in the target trial is similar to the intention-to-treat analysis except participants are censored if and when they deviate from their assigned treatment strategy. To adjust for the potential selection bias induced by this censoring, we need to adjust for prognostic factors that are associated with non-adherence. Given the available information in SWEDEHEART and linked registers, we select the following post-baseline (time-varying) prognostic factors: diagnosis of renal disease; and dispensation of angiotensin 2 receptor blockers, ACE inhibitors, calcium channel blockers, diuretics, nitrates, or diabetes treatments. These variables are used to estimate time-varying IP weights for all individuals [17].

Because adherence to the assigned treatment may vary substantially between the randomized trial and the routine clinical setting, the intention to treat effects estimated in each setting may differ even if both estimates are correct. Though the index trial protocol does not consider estimating the per-protocol effect as a primary aim of the study, we will aim at doing so when its data become available.

### The observational emulation of the target trial

We used observational data from the SWEDEHEART registry and linked registers to emulate the target trial. SWEDEHEART includes all patients hospitalized for acute coronary syndrome or undergoing coronary or valvular intervention for any indication in all relevant hospitals across Sweden [9]. The registry was created by merging 4 existing cardiovascular health-care quality registries in 2009: the Register of Information and Knowledge About Swedish Heart Intensive Care Admissions (RIKSHIA), the Swedish Coronary Angiography and Angioplasty Registry (SCAAR), the Swedish Heart Surgery Registry, and the National Registry of Secondary Prevention (SEPHIA). SWEDEHEART is also regularly linked to the Swedish Total Population Register; the Swedish National Patient Register, which records all primary and secondary diagnoses and procedures from inpatient hospitalizations and outpatient specialist care visits across Sweden; the Swedish Cause of Death Register, which records all deaths and causes of death; and the Prescribed Drug Register, which collects information on all prescribed and dispensed medications [18–20].

Each component of the protocol of the target trial was emulated as closely as possible using the SWEDEHEART registry data (see Table 1 for details). Eligible individuals were “assigned” to the beta blocker group if they received a prescription of beta blockers within 30 days of angiography, and to the no beta blocker group if they did not receive such prescription. The period of eligibility in the emulation was longer compared with the index trial as many individuals are prescribed beta blockers in the community soon after hospital discharge in routine practice. Individuals with an event in the 30 days following angiography were excluded, which is unlikely to introduce bias because an acute effect of beta blockers is not expected and because individuals at very high risk of an imminent outcome will not be included in the index trial.

In the beta blocker group, the daily treatment dose was assumed to be 100 mg for metoprolol and 5 mg for bisoprolol as the daily treatment dose in Prescribed Drug Register is only correct for primary indication of treatment, and secondary prevention after myocardial infarction is not the primary indication for beta blockers. The intended length of each prescribed dispensation was then calculated from the number of pills and pill dose divided by daily dose. The REDUCE-AMI protocol does not specify a definition of adherence, so we proposed the definition of adherence to be a gap of less than 180 days between the end date of one dispensation and the following dispensation, unless this was preceded by a contraindication, in the beta blocker group, and continuing to not dispense beta blockers, unless this was preceded by a new indication, in the no beta blocker group.

We assumed that assignment (first prescription) was random within levels of the baseline covariates: hospital, year of index, age, sex, smoking status, hypertension, diabetes, previous myocardial infarction, previous stroke, previous percutaneous coronary intervention, history of coronary heart failure, previous cardiac surgery, renal disease, other serious diseases, angiotensin 2 receptor blockers, ACE inhibitors, calcium channel blockers, diuretics, nitrates, diabetes treatments, infarction type, cardiopulmonary resuscitation before hospital, thrombolysis before hospital, cardiogenic shock, electrocardiogram (ECG) rhythm, ECG QRS annotation, ECG ST- & T-wave changes, percutaneous coronary intervention, angiography finding, stenosis class, proportion stenosis, IV beta blockers, IV diuretics, IV inotropic drugs, IV nitrates, heart rate, systolic blood pressure, diastolic blood pressure, LDL cholesterol, HDL cholesterol, creatinine, body mass index (see Supplementary Table 1). The outcomes were the same as the target trial and also identified in the Swedish Total Population Register and SWEDEHEART like REDUCE-AMI, but data were not available on migration.

The intention-to-treat analysis (i.e., effect of first prescription) was the same as for the target trial with all baseline covariates adjusted for. The per-protocol analysis was the same as the target trial. For primary analyses, missing data for continuous covariates were imputed using the median of all non-missing instances, and missing categories were included for categorical variables. Non-parametric bootstrapping with 500 samples was used to calculate 95% confidence intervals.

### Sensitivity analyses

We assessed the robustness of our effect estimates to several analytic decisions. For the intention-to-treat analysis, we aimed to understand: (1) the impact of different modelling assumptions by including baseline covariates in the pooled logistic regression model instead of using IP weights; (2) the sensitivity of the results to emulation of the eligibility criteria “no indication for beta blockers other than as secondary prevention” by (2a) modifying the eligibility criteria to not exclude individuals with a beta blocker before baseline (and additionally adjusting for prior beta blockers recorded in either SWEDEHEART or in the prescribed drug register in the prior 3 years), and (2b) reformulating the operationalization of prior use of beta blockers to be only those with registered beta blocker use on hospital admission in SWEDHEART rather than in both SWEDHEART and the prescribed drug register (and additionally adjusting for prior beta blockers in the prescribed drug register in the prior 3 years); (3) the sensitivity of the results to the missing data assumptions by (3a) carrying out a complete case analysis and (3b) categorizing continuous variables with a “missing” category (categories specified in Supplementary Table 1); and (4) the impact of the decision to extend baseline through 30 days following angiography on the risk of misalignment of eligibility, treatment assignment, and start of follow up by using a cloning and censoring approach (full details in “Appendix”) [21, 22]. For the per-protocol analysis, we reduced the time allowed between prescription dispensations for the definition of continuous treatment in the beta blocker group to 90 days rather than 180 days to understand if having larger gaps between dispensations affected results.

## Results

**Fig. 1:**
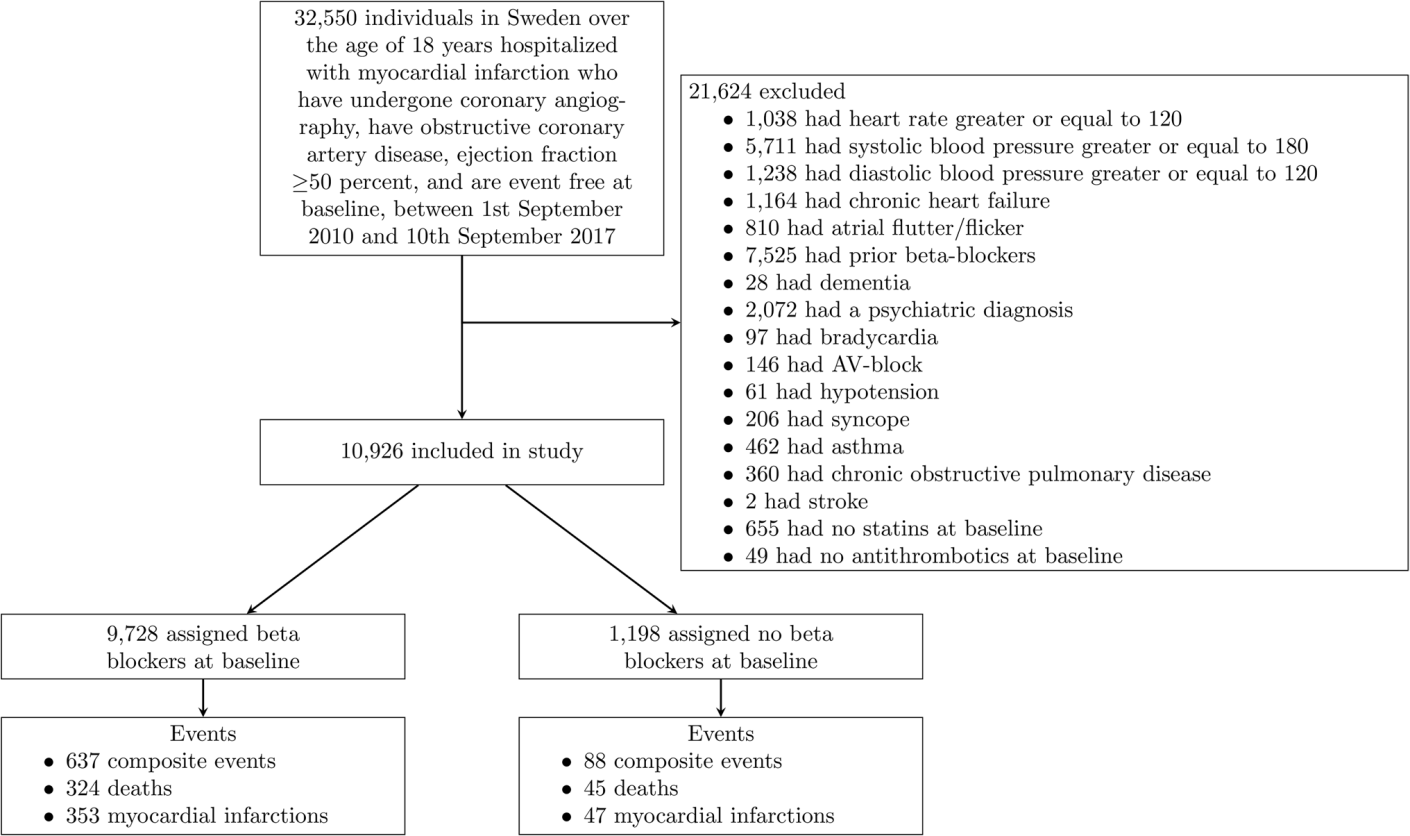
Flowchart for selection of eligible individuals into an emulation of a target trial of beta blockers versus no beta blockers in individuals with myocardial infarction with preserved ejection fraction in Sweden, 2011–2017

Figure 1 shows a flowchart of selection into the target trial emulation, and Table 2 shows the baseline characteristics for the 10,926 eligible individuals. Compared with those assigned to no beta blockers (1,198 individuals), those assigned to beta blockers (9,728 individuals) were, on average, younger, and more likely to smoke and to present with NSTEMI with a higher proportion of coronary stenosis, and less likely to have prior cardiac diseases. They also had higher heart rate and blood pressure.

**Table 2:** Baseline characteristics of eligible individuals for an emulation of a target trial of beta blockers versus no beta blockers in Sweden, 2011–2017 (continued in Supplementary Table 5)

	Beta blockers	No beta blockers	Missing	SMD	SMD after IP weighting
	9728	1198			
*Characteristics and prior diagnoses*					
Age	64.0 [56.0, 72.0]	67.0 [58.0, 74.0]	0	0.181	0.031
Female	2060 (21.2)	235 (19.6)	0	0.039	0.041
Smoking status			1.7	0.126	0.034
Never smoker	3948 (41.3)	535 (45.4)			
Ex-smoker (> 1 month)	3387 (35.4)	427 (36.2)			
Smoker	2226 (23.3)	216 (18.3)			
Hypertension	2968 (30.6)	390 (32.6)	0.2	0.044	0.037
Diabetes	1065 (11.0)	108 (9.0)	0.1	0.065	0.042
Myocardial infarction	388 (4.0)	80 (6.7)	0.1	0.12	0.028
Stroke	223 (2.3)	43 (3.6)	0.1	0.077	0.057
Percutaneous coronary intervention	271 (2.8)	76 (6.3)	0.1	0.171	0.036
Cardiac surgery	83 (0.9)	27 (2.3)	0.1	0.113	0.044
Renal disease	131 (1.3)	14 (1.2)	0	0.016	0.007
Cancer	54 (0.6)	8 (0.7)	0	0.191	0.026
*Presentation*					
NSTEMI	5492 (56.5)	786 (65.6)	0	0.189	0.012
Cardiopulmonary resuscitation	159 (1.7)	5 (0.4)	0.9	0.122	0.098
Thrombolysis	41 (0.4)	4 (0.3)	0.1	0.014	0.019
Cardiogenic shock	38 (0.4)	9 (0.8)	0.2	0.048	0.031
ECG ryhthm			0.2	0.068	0.047
Sinus	9620 (99.1)	1176 (98.3)			
Atrial flicker/flutter	0 (0.0)	0 (0.0)			
Other	88 (0.9)	20 (1.7)			
ECG QRS annotation			0.9	0.066	0.046
Normal	8079 (83.8)	970 (81.5)			
Pacemaker	6 (0.1)	1 (0.1)			
Left bundle branch block	100 (1.0)	13 (1.1)			
Pathological Q-wave	601 (6.2)	82 (6.9)			
Right bundle branch block	256 (2.7)	41 (3.4)			
Other	597 (6.2)	83 (7.0)			
ECG ST- & T-wave changes			0.4	0.234	0.043
Normal	2459 (25.4)	362 (30.3)			
ST-elevation	4142 (42.8)	391 (32.7)			
ST-depression	1602 (16.5)	190 (15.9)			
Pathological T-wave	897 (9.3)	149 (12.5)			
*During hospitalization*					
Percutaneous coronary intervention	8887 (91.4)	1135 (94.7)	0	0.133	0.016
Angiography finding			0	0.136	0.075
1 vessel, not left main	5126 (52.7)	680 (56.8)			
2 vessels, not left main	2685 (27.6)	340 (28.4)			
3 vessels not left main	1454 (14.9)	136 (11.4)			
Left main + 1 vessel	75 (0.8)	7 (0.6)			
Left main + 2 vessels	136 (1.4)	11 (0.9)			
Left main + 3 vessels	231 (2.4)	20 (1.7)			
Left main	21 (0.2)	4 (0.3)			
Stenosis class			10.3	0.087	0.050
A	550 (6.3)	84 (7.6)			
B1	2538 (29.2)	329 (29.7)			
B2	2876 (33.1)	378 (34.1)			
C	1324 (15.2)	161 (14.5)			
B1 bifurcation	459 (5.3)	55 (5.0)			
B2 bifurcation	686 (7.9)	68 (6.1)			
C bifurcation	261 (3.0)	32 (2.9)			
Proportion stenosis			4.5	0.131	0.084
< 70%	210 (2.3)	43 (3.7)			
70–89%	1391 (15.0)	200 (17.2)			
90–99%	3497 (37.7)	456 (39.3)			
100%	4171 (45.0)	462 (39.8)			
Intravenous beta blockers	749 (7.7)	18 (1.5)	0	0.299	0.111
Intravenous diuretics	325 (3.3)	24 (2.0)	0	0.083	0.052
Intravenous inotropic drugs	118 (1.2)	12 (1.0)	0	0.02	0.053
Intravenous nitrates	633 (6.5)	51 (4.3)	0.1	0.1	0.051
*Concomitant medications*					
Angiotensin 2 receptor blockers	1747 (18.0)	231 (19.3)	0	0.034	0.063
ACE inhibitors	6784 (69.7)	713 (59.5)	0	0.215	0.016
Calcium channel blockers	1657 (17.0)	257 (21.5)	0	0.112	0.023
Diuretics	1212 (12.5)	161 (13.4)	0	0.029	0.041
Nitrates	8490 (87.3)	1050 (87.6)	0	0.011	0.022
Diabetes treatment	1130 (11.6)	107 (8.9)	0	0.089	0.006
*Measurements*					
Heart rate	72.0 [63.0, 83.0]	63.0 [55.0, 74.0]	0	0.603	0.088
Systolic blood pressure (mm/Hg)	148.0 [130.0, 160.0]	145.0 [130.0, 160.0]	0	0.069	0.004
Diastolic blood pressure (mm/Hg)	85.0 [75.0, 95.0]	80.0 [73.0, 90.0]	0	0.218	0.034
LDL cholesterol (mmol/L)	3.3 [2.7, 4.0]	3.3 [2.6, 4.0]	15.5	0.033	0.029
HDL cholesterol (mmol/L)	1.2 [1.0, 1.4]	1.2 [1.0, 1.4]	13.7	0.084	0.016
Creatinine (µmol/L)	78.0 [68.0, 90.0]	81.0 [70.0, 93.0]	4.4	0.148	0.005
Body mass index (kg/m^2^)	27.0 [25.0, 30.0]	27.0 [24.0, 29.0]	2.4	0.124	0.014

*SMD* standardized mean difference, *IP* Inverse probability

### Intention-to-treat effect: the effect of prescription versus no prescription of beta blockers at baseline

**Fig. 2:**
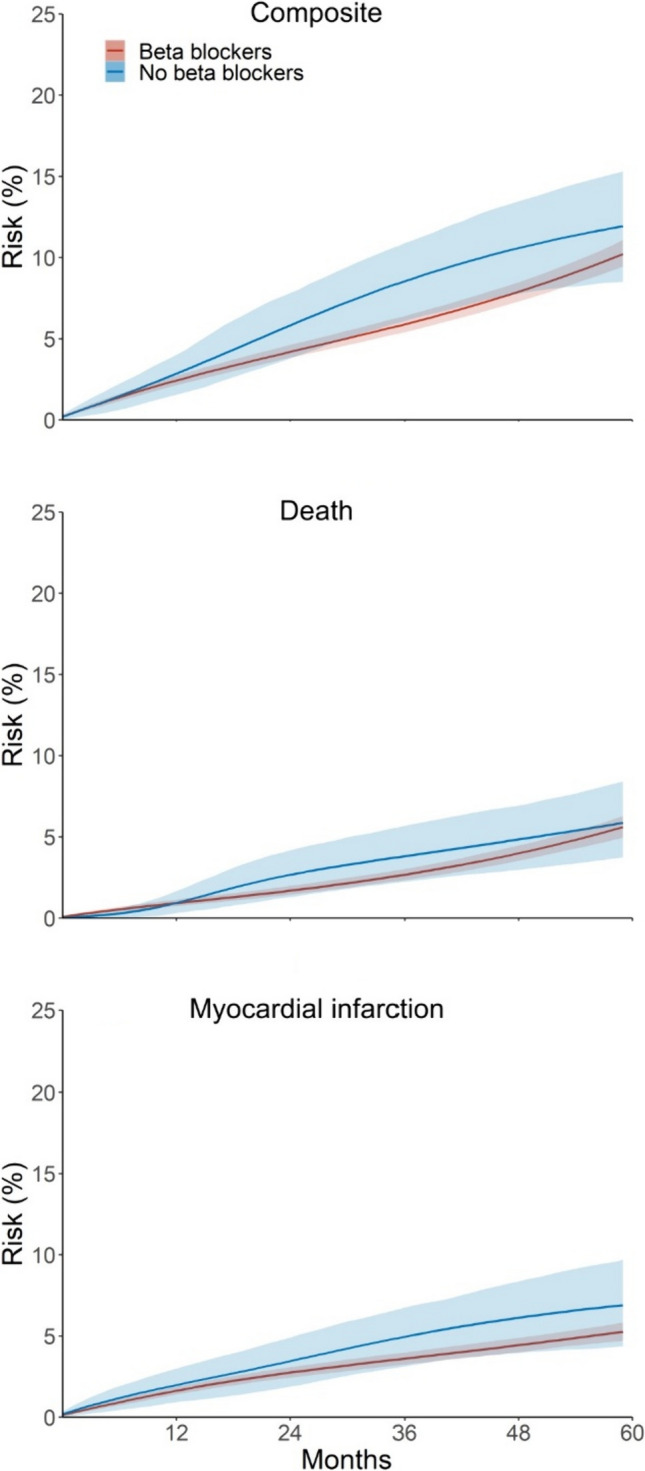
Estimated risk of death and myocardial infarction under beta blockers versus no beta blockers. Intention-to-treat analysis of an emulated target trial in individuals with myocardial infarction with preserved ejection fraction in Sweden, 2011–2017 (shaded intervals represent limits of the pointwise 95% CIs)

Median follow up was 41 months in the beta blocker group and 36 months in the no beta blocker group. The estimated 5-year risks of the composite outcome were 10.2% (9.4%, 11.1%) under beta blockers and 11.9% (8.5%, 15.3%) under no beta blockers, which results in a risk difference of − 1.7% (− 5.5%, 1.9%), a risk ratio of 0.86 (0.64, 1.23) (Table 3 and Fig. 2), and an average hazard ratio by 5 years of 0.78 (0.59, 1.12) (Table 4). We also show the hazard ratio with follow up time terminated at 3 and 4 years in Table 4.

**Table 3:** Estimated risks of death and myocardial infarction by 5 years in an emulation of a target trial of beta blockers versus no beta blockers in Sweden, 2011–2017

Outcome	Beta blockers		No beta blockers		Risk difference (95% CI)	Risk ratio (95% CI)
	Events	Risk, % (95% CI)	Events	Risk, % (95% CI)		
*Intention-to-treat*^*a*^						
Composite	637	10.2 (9.4, 11.1)	88	11.9 (8.5, 15.3)	− 1.7 (− 5.5, 1.9)	0.86 (0.64, 1.23)
Death	324	5.6 (4.9, 6.3)	45	5.9 (3.7, 8.4)	− 0.3 (− 2.9, 1.9)	0.96 (0.65, 1.48)
Myocardial infarction	353	5.3 (4.7, 5.8)	47	6.9 (4.4, 9.7)	− 1.6 (− 4.5, 1.0))	0.76 (0.53, 1.22)
*Per-protocol*^*a,b*^						
Composite	523	10.2 (9.3, 11.2)	75	11.0 (7.6, 14.4)	− 0.8 (− 4.5, 2.8)	0.92 (0.69, 1.37)
Death	259	5.6 (4.9, 6.5)	41	5.7 (3.5, 8.5)	− 0.1 (− 2.8, 2.2)	0.99 (0.67, 1.60)
Myocardial infarction	295	5.2 (4.6, 5.9)	37	5.9 (3.3, 8.5)	− 0.7 (− 3.3, 2.1)	0.88 (0.60, 1.59)

^a^Adjusted for baseline variables in Tables 2 and Supplementary Table 1

^b^Further adjusted for time-varying variables in Supplementary Table 1

**Table 4:** Estimated hazard ratios of the composite outcome for beta blockers versus no beta blockers by 3, 4, and 5 years in an emulation of a target trial in Sweden, 2011–2017

Time of follow up termination	Events		Hazard ratio (95% CI)
	Beta blockers	No beta blockers	
*Intention-to-treat*^*a*^			
3 years	454	65	0.75 (0.54, 1.12)
4 years	545	83	0.69 (0.51, 0.99)
5 years	637	88	0.78 (0.59, 1.12)
*Per-protocol*^*a,b*^			
3 years	390	56	0.79 (0.57, 1.25)
4 years	457	70	0.74 (0.56, 1.11)
5 years	523	75	0.84 (0.64, 1.24)

^a^ Adjusted for baseline variables in Tables 2 and Supplementary Table 1

^b^Adjusted for time-varying variables in Supplementary Table 1

For each component of the composite outcome, the estimated 5-year risks of death were 5.6% (4.9%, 6.3%) under beta blockers and 5.9% (3.7%, 8.4%) under no beta blocker, which results in a risk difference of − 0.3% (− 2.9%, 1.9%), a risk ratio of 0.96 (0.67, 1.45), and an average hazard ratio by 5 years of 0.85 (0.59, 1.38); the estimated 5-year risks of myocardial infarction were 5.3% (4.7%, 5.8%) under beta blockers and 6.9% (4.4%, 9.7%) under no beta blockers, which results in a risk difference of − 1.6% (− 4.5%, 1.0%), a risk ratio of 0.76 (0.53, 1.22), and an average hazard ratio by 5 years of 0.75 (0.53, 1.21).

Results in sensitivity analyses were broadly similar to those from the main analysis for the composite outcome (Appendix Table 2).

### Per-protocol effect: the effect under full adherence to the treatment strategy in the protocol

Adherence was 74% in the beta blocker and 91% in the no beta blockers group. After censoring at non-adherence to the assigned treatment strategy but before IP weighting, median follow up was 29 months in the beta blocker group and 31 months in the no beta blocker group. The estimated 5-year risks of the composite outcome were 10.2% (9.3%, 11.2%) under beta blockers and 11.0% (7.6%, 14.4%) under no beta blockers, which results in a risk difference of − 0.8% (− 4.5%, 2.8%), a risk ratio of 0.92 (0.69, 1.37) (Table 3 and Supplementary Fig. 1), and an average hazard ratio by 5 years of 0.84 (0.64, 1.24) (Table 4). We, again, show the hazard ratios with follow up terminated at 3 and 4 years in Table 4.

For each component of the composite outcome, the estimated 5-year risks of death were 5.6% (4.9%, 6.5%) under beta blockers and 5.7% (3.5%, 8.5%) under no beta blockers, which results in a risk difference of − 0.1% (− 2.8%, 2.2%), a risk ratio of 0.99 (0.67, 1.60), and an average hazard ratio by 5 years of 0.85 (0.58, 1.36); the estimated 5-year risks of myocardial infarction were 5.2% (4.6%, 5.9%) under beta blockers and 5.9% (3.3%, 8.5%) under no beta blockers, which results in a risk difference of − 0.7% (− 3.3%, 2.1%), a risk ratio of 0.88 (0.60, 1.59), and an average hazard ratio by 5 years 0.86 (0.60, 1.53).

Results in the sensitivity analyses were broadly similar to the main analysis for the composite outcome (Supplementary Table 3).

## Discussion

We used observational data from SWEDEHEART and linked registers to emulate a target trial with a protocol similar to that of REDUCE-AMI, an ongoing trial which is also embedded in SWEDEHEART. Had everyone adhered to the treatment strategy as specified in the target trial protocol, we estimated a reduction in the 5-year risk of death or myocardial infarction of 0.8 percentage points for beta blockers compared with no beta blockers. However, effects ranging from a reduction of 4.5 percentage points to an increase of 2.8 percentage points are compatible with our data under conventional statistical criteria. Once results of REDUCE-AMI are published, we will compare results of our observational analysis against those from the trial to understand if our emulation was successful.

Randomized trials and observational studies differ, of course, in the treatment assignment procedure, which is randomized in the trials but not in the observational data. Also, randomized and observational analyses that ask a similar question often differ with respect to eligibility criteria, causal contrast, and measurement of treatment or outcomes. We will now consider these differences and their potential impact when comparing results between REDUCE-AMI and our observational emulation.

Despite coming from the same registry, the study populations of the target trial emulation and the index trial will be slightly different for three reasons. First, the target trial emulation cannot exclude individuals who would not enrol in a randomized trial. Because generally, healthier individuals of higher socioeconomic status agree to enrol in trials, we expect index trial participants will have less severe disease and a lower comorbidity burden than those in the target trial emulation [23]. Second, the target trial emulation cannot fully replicate eligibility criteria that are vaguely articulated for the index trial. Specifically, the index trial will exclude individuals if they have an “indication for beta blockers other than as secondary prevention according to the treating physician” (but prior beta blocker use is not an explicit exclusion) [8]. To operationalize this ambiguous eligibility criterion, the target trial excludes anyone with prior use of any beta blocker (recorded in SWEDHEART or the prescribed drug register in the prior 3 year) or with characteristics that would mean they were indicated for a reason other than secondary prevention (high heart rate, high blood pressure, heart failure, atrial flutter). When we included those with prior beta blocker use in a sensitivity analysis, the 5-year risk was higher in both groups and the effect estimates was attenuated, compared with the main analysis. Third, the target trial emulation was restricted to individuals who received statins and anti-thrombotic treatment at baseline, which occurred in 94% of otherwise eligible individuals. This eligibility criterion excludes individuals who are ineligible for statins or anti-thrombotics (e.g.,  individuals that do not tolerate statins or have very high bleeding risk) and who perhaps did not receive these treatments because of poor post-myocardial infarction prognosis. Subgroup analyses may be needed to understand if a different composition of the study populations explains different effect estimates between the index trial and the target trial emulation.

REDUCE-AMI will estimate an intention-to-treat effect, which is the effect of assignment to one of the treatment strategies under the level of adherence observed in the trial over follow-up. The magnitude of the intention-to-treat effect depends on the type of assignment and adherence. Therefore, estimates of the intention-to-treat effect in the index trial and the observational emulation of the target trial may differ because of differences in assignment (a randomized prescription in the trial versus a routine prescription in the observational data) or adherence (still unknown in the trial) [24]. A more comparable contrast is the per protocol effect [25], which we plan to estimate once trial data become available (as part of a fully harmonized analysis, including the handling of competing events).

A key advantage of our study is that outcome definitions should be the same as those used in REDUCE-AMI as both studies use routinely collected data from Swedish registers (SWEDEHEART and the Total Population Register) to identify individuals with an outcome. Variation would be possible if, the coding of myocardial infarction had changed between the time of the trial and observational analysis, but this is not the situation in our example.

The main effect measure in REDUCE-AMI will be the hazard ratio from a Cox model, that is a weighted average of the time-varying hazard ratios during the follow-up. Because this average hazard depends on the length of follow-up and the distribution of censoring [26], hazard ratio estimates in the index trial and the emulated target trial may differ. To ameliorate this problem, we report average hazard ratios when follow up is terminated at three, four, and five years in Table 4. More comparable effect measures are the risk difference and risk ratio at a fixed time of follow-up (e.g., five years), which we plan to estimate in both studies once trial data become available.

The observational data used to emulate the target trial had detailed clinical information on the index myocardial infarction required for eligibility. However, there were still data limitations that restricted the emulation, such as no access to primary care records. A consequence of this is that we may have missed individuals with other beta blocker indications or with a beta blocker contraindication. Additionally, without continuously updated information on lab values and diagnoses that did not require a hospital visit for the duration of follow up, we miss potential prognostic factors associated with nonadherence to the treatment protocol. This could be a source of residual bias in our per protocol analyses.

In summary, we have designed a target trial with a protocol similar to REDUCE-AMI, then emulated the target trial using observational data from the same population in which REDUCE-AMI participants are recruited. Randomized trials cannot answer all important clinical questions, and observational data can be used to complement these trials and fill evidence gaps; benchmarking an observational analysis against a randomized trial then using the observational data to ask additional questions is one way to achieve this. Prospective benchmarking shifts the investigator focus away from an endeavour to use observational data to estimate similar results as a completed randomized trial, to a systematic attempt to align the design and analysis of the trial and the observational analysis.

### Supplementary Information

Below is the link to the electronic supplementary material.

Supplementary file1 (DOCX 183 KB)

## Data Availability

All analysis code is available at: https://github.com/tonymatthews/reduce.
