# Supplementary material for: Prospective benchmarking of an observational analysis in the SWEDEHEART registry against the REDUCE-AMI randomized trial
Source: Eur J Epidemiol. 2024 May 8;39(4):349–61. doi: 10.1007/s10654-024-01119-3 (PMC11101517; doi:10.1007/s10654-024-01119-3)
Supplement: Supplementary file 1 — Supplementary file1 (DOCX 183 KB) [file 10654_2024_1119_MOESM1_ESM.docx]

Supplementary Materials

**Contents**

[Appendix – Sensitivity analysis to address potential misalignment of eligibility, treatment assignment, and start of follow up 2](#_Toc165142153)

[Supplementary Table 1 - Covariate definitions 3](#_Toc165142154)

[Supplementary Table 2 - Sensitivity analyses for intention-to-treat analysis with composite outcome 7](#_Toc165142155)

[Supplementary Table 3 - Sensitivity analyses for per-protocol analysis with composite outcome 8](#_Toc165142156)

[Supplementary Table 4 – ICD-10 and ATC codes used to define diagnoses and treatments for eligibility criteria and treatment strategies 9](#_Toc165142157)

[Supplementary Table 5 - Baseline characteristics of eligible individuals for an emulation of a target trial of beta blockers vs. no beta blockers in Sweden, 2010-2017, continued from Table 2 10](#_Toc165142158)

[Supplementary Figure 1 - Estimated risk of death and myocardial infarction under beta blockers versus no beta blockers. Per protocol analysis of an emulated target trial in individuals with myocardial infarction with preserved ejection fraction in Sweden, 2011–2017 (shaded intervals represent limits of the pointwise 95% CIs) 12](#_Toc165142159)

## Appendix – Sensitivity analysis to address potential misalignment of eligibility, treatment assignment, and start of follow up

*Potential problems with the simplified main analysis*

In our primary analysis, baseline (time zero) is defined as the 30-day period after coronary angiography following myocardial infarction. In this 30-day period, we assess if individuals reach all eligibility criteria, and if they are prescribed a beta blocker or not. By definition, individuals must also be event free at baseline, so anyone that dies or has a recurrent myocardial infarction during this period is excluded.

In our setting, this simplified approach allows us to substantially speed up the computational time for the analysis without introducing any meaningful bias (see below). However, this approach is not generally recommended because of two potential biases. First, individuals may be prescribed beta blockers prior to reaching all eligibility criteria. As per the eligibility criteria of our target trial, an individual must be on statins and antithrombotic medication, but not have prior beta blocker use to be eligible. For example, if an individual is prescribed beta blockers on day 3 after angiography, but a statin and antithrombotic on day 10, they are technically not eligible, but will be included in our primary study design. Second, an individual may not be assigned to a treatment group at the exact date they became eligible. As the treatment strategies are beta blockers versus no beta blockers within 30 days of angiography, we must wait until 30 days have elapsed until we know if an individual is assigned to the no beta blocker arm; if they are prescribed a beta blocker before the 30 days elapse, they are assigned to the beta blocker arm at time of prescription. This could induce immortal time as we exclude some individuals that have an event after becoming eligible if the event happens within the 30 days after angiography. Among the individuals that were not already prescribed beta blockers before an event in the first 30 days, we do not know if they would go on to be prescribed or if they would be assigned to the no beta blocker group.

*A more refined analysis that avoids these problems*

To overcome the potential misalignment of eligibility, treatment assignment, and start of follow up, described above, we identified the exact day individuals reached all eligibility criteria, which could have been up to 30 days after date of angiography (if, for example, they were prescribed a statin and antithrombotic later). Then, to prevent immortal time, we cloned all individuals on the day they met all eligibility criteria; one clone was assigned to the beta blocker group and the other to the no beta blocker group. We started follow up on the first day an individual became eligible. Then, clones assigned to the no beta blocker group were censored on the day they were prescribed beta blocker (if they were prescribed one during the 30-day period after angiography), and individuals assigned to the beta blocker group were censored 30 days after angiography if they were not prescribed beta blockers by that time. Follow up was then continued as normal until an event occurred, 5 years elapsed, or the individual was lost to follow up.

## Supplementary Table 1 - Covariate definitions

| **Covariate** | **Register** | **Definition** | **Values** | **Form** | **Baseline/time-updated** |
| --- | --- | --- | --- | --- | --- |
| **Hospital** | RIKSHIA | Hospital where individual was admitted |  | Categorical | Baseline |
| **Year of index** | RIKSHIA | Year an individual has their index date | 2012-2017 | Categorical | Baseline |
| **Age** | RISKHIA | Age at hospital admission | Sensitivity: <55 \| 56-70 \| 71-85 \| 86+ \| NA | Main: Restricted cubic spline with five knots  Sensitivity: Categorial | Baseline |
| **Sex** | RIKSHIA | Sex | 1 = Male \| 2 = Female | Categorical | Baseline |
| **Smoking status** | RIKSHIA | Self reported smoking at admission (also applies to hookah) | 0 = Never smoker \| 1 = Ex smoker> 1 month \| 2 = Smoker \| 9 = Unknown | Categorical | Baseline |
| **Hypertension** | RIKSHIA | Drug treatment for hypertension at admission or any time before | 0 = No \| 1 = Yes \| 9 = Unknown | Categorical | Baseline |
| **Diabetes** | RIKSHIA | Diagnosis of diabetes any time before admission, regardless of treatment | 0 = No \| 1 = Yes \| 9 = Unknown | Categorical | Baseline |
| **Previous myocardial infarction** | RIKSHIA | Diagnosis of myocardial infarction as any time before admission, either through documentation in patient record or self report. Also Specify if had silent myocardial infarction that the patient is unaware of, but which has been shown on an ECG. | 0 = No \| 1 = Yes \| 9 = Unknown | Categorical | Baseline |
| **Previous stroke** | RIKSHIA | Diagnosis of ischemic stroke or bleeding at any time before admission, not including TIA, through documentation in patient record | 0 = No \| 1 = Yes \| 9 = Unknown | Categorical | Baseline |
| **Previous percutaneous coronary intervention** | RIKSHIA | Specify whether the patient has previously undergone a PCI (Percutaneous Coronary Intervention) of any type before the current insertion (balloon dilation, atherectomy, stent or other). Retrieved from previous registration | 0 = No \| 1 = Yes \| 9 = Unknown | Categorical | Baseline |
| **Previous cardiac surgery** | RIKSHIA | Specify whether the patient has undergone open heart surgery before this hospitalization | 0 = No \| 1 = Yes \| 9 = Unknown | Categorical | Baseline |
| **Renal disease** | Patient register | ICD-10 code (N17-19) in the inpatient or outpatient register as a primary or secondary diagnosis within 3 years of assignment | 0 = No \| 1 = Yes | Categorical | Both |
| **Other serious diseases** | RIKSHIA | Specify whether the patient is suffering from any other serious illness which means that recommended treatment / action cannot be given / performed | 0 = No \| 1 = Cancer \| 8 = Other | Categorical | Baseline |
| **Angiotensin 2 receptor blockers** | Prescribed drug register | Dispensation ATC code (C09C, C09D) in the prescribed drug register within 3 years of assignment | 0 = No \| 1 = Yes | Categorical | Both |
| **ACE inhibitors** | Prescribed drug register | Dispensation ATC code (C09A, C09B) in the prescribed drug register within 3 years of assignment | 0 = No \| 1 = Yes | Categorical | Both |
| **Calcium channel blockers** | Prescribed drug register | Dispensation ATC code (C08) in the prescribed drug register within 3 years of assignment | 0 = No \| 1 = Yes | Categorical | Both |
| **Diuretics** | Prescribed drug register | Dispensation ATC code (C03) in the prescribed drug register within 3 years of assignment | 0 = No \| 1 = Yes | Categorical | Both |
| **Nitrates** | Prescribed drug register | Dispensation ATC code (C01DA) in the prescribed drug register within 3 years of assignment | 0 = No \| 1 = Yes | Categorical | Both |
| **Diabetes treatments** | Prescribed drug register | Dispensation ATC code (A10) in the prescribed drug register within 3 years of assignment | 0 = No \| 1 = Yes | Categorical | Both |
| **Infarction type** | RIKSHIA | Whether the patient is perceived by the treating physician as a STEMI or non-STEMI patient | 1 = STEMI \| 2 = NSTEMI | Categorical | Baseline |
| **CPR before hospital** | RIKSHIA | Specify whether CPR (even defibrillation only) was performed prior to arrival at hospital | 0 = No \| 1 = Yes \| 9 = Unknown | Categorical | Baseline |
| **Thrombolysis before hospital** | RIKSHIA | Specify whether thrombolysis was given prior to arrival in hospita | 0 = No \| 1 = Yes \| 9 = Unknown | Categorical | Baseline |
| **Cardiogenic shock** | RIKSHIA | Cardiogenic shock on arrival to hospital | 0 = No \| 1 = Yes \| 9 = Unknown | Categorical | Baseline |
| **ECG rhythm** | RIKSHIA | ECG rhythm with regards to the ECG that is the basis for decision making for admission | 1 = Sinus \| 2 = Ability flicker / flutter \| 8 = Other \| 9 = Unknown | Categorical | Baseline |
| **ECG QRS annotation** | RIKSHIA | QRS annotation with regards to the ECG that is the basis for decision making for admission | 1 = Normal \| 2 = Pacemaker \| 3 = Left branch block \| 4 = Patthological Q wave \| 5 = Right branch block \| 8 = Other \| 9 = Unknown | Categorical | Baseline |
| **ECG ST- & T-wave changes** | RIKSHIA | ST- & T-wave changes with regards to the ECG that is the basis for decision making for admission | 1 = Normal \| 2 = ST raise \| 3 = ST reduction \| 4 = Pathological T-wave \| 8 = Other \| 9 = Unknown | Categorical | Baseline |
| **Percutaneous coronary intervention** | RIKSHIA | Specify whether the patient underwent percutaneous coronary intervention | 0 = No \| 1 = Yes \| 9 = Unknown | Categorical | Baseline |
| **Angiography finding** | SCAAR | Finding from agiography | 2 = 1 vessel not left main \| 3 = 2 vessels not left main \| 4 = 3 vessels not left main \| 5 = left main + 1 vessel \| 6 = left main + 2 vessels \| 7 = left main + 3 vessels \| 8 = left main | Categorical | Baseline |
| **Stenosis class** | SCAAR | Stenosis according to J Am Coll Cardiol 1988: 12 (2): 529-45. | 1 = A \| 2 = B1 \| 3 = B2 \| 4 = C \| 5 = B1 Bifurcation \| 6 = B2 Bifurcation \| 7 = C Bifurcation \| 9 = Other | Categorical | Baseline |
| **Proportion stenosis** | SCAAR | Proportion stenosis in artery with highest level of stenosis (if more than one) | 1 = 0% \| 2 = <50% \| 3 = 50-69% \| 4 = 70-89% \| 5 = 90-99% \| 6 = 100% \| 9 = Unkown | Categorical | Baseline |
| **IV beta blockers** | RIKSHIA | Specify whether intravenous beta blcokers were given during the hospitalization, most recent instance during hospitalization | 0 = No \| 1 = Yes \| 9 = Unknown | Categorical | Baseline |
| **IV diuretics** | RIKSHIA | Specify whether intravenous diuretics were given during the hospitalization, most recent instance during hospitalization | 0 = No \| 1 = Yes \| 9 = Unknown | Categorical | Baseline |
| **IV inotropic drugs** | RIKSHIA | Specify whether intravenous intropic drugs were given during the hospitalization, most recent instance during hospitalization | 0 = No \| 1 = Yes \| 9 = Unknown | Categorical | Baseline |
| **IV nitrates** | RIKSHIA | Specify whether intravenous nitrates were given during the hospitalization, most recent instance during hospitalization | 0 = No \| 1 = Yes \| 9 = Unknown | Categorical | Baseline |
| **Heart rate** | RIKSHIA | Heart rate (beats per minute). It should be the first heart rate noted by healthcare professionals. | Sensitivity: <59 \| 60-99 \| 100+ \| NA | Main: Restricted cubic spline with five knots  Sensitivity: Categorial | Baseline |
| **Systolic blood pressure** | RIKSHIA | Systolic blood pressure. It should be the first blood pressure noted by healthcare professionals, which includes primary care physicians / ambulance and emergency room personnel | Sensitivity: <119\| 120-139 \| 140+ \| NA | Main: Restricted cubic spline with five knots  Sensitivity: Categorial | Baseline |
| **Diastolic blood pressure** | RIKSHIA | Diastolic blood pressure. It should be the first blood pressure noted by healthcare professionals, which includes primary care physicians / ambulance and emergency room personnel | Sensitivity: <79 \| 80-89 \| 90+ \| NA | Main: Restricted cubic spline with five knots  Sensitivity: Categorial | Baseline |
| **LDL cholesterol** | RIKSHIA | LDL in mmol/L is calculated primarily as total cholesterol - HDL - (0.45 x triglycerides) provided these values ​​are present and that the value of triglycerides is <= 4.5 mmol / L. If the calculation does not yield any results, the manually entered LDL value is used if available. Negative values ​​are set to missing. | Sensitivity: <3.3 \| 3.4-4.8 \| 4.9+ \| NA | Main: Restricted cubic spline with five knots  Sensitivity: Categorial | Baseline |
| **HDL cholesterol** | RIKSHIA | HDL in mmol/L, should be taken during first day of care of heart attack | Sensitivity: <0.9 \| 1.0-1.5 \| 1.5+ \| NA | Main: Restricted cubic spline with five knots  Sensitivity: Categorial | Baseline |
| **Creatinine** | RIKSHIA | Creatinine, takes the first lab value (non missing) during admission. | Sensitivity: <44 \| 45-59 \| 60-89 \| 90+ \| NA | Main: Restricted cubic spline with five knots  Sensitivity: Categorial | Baseline |
| **BMI** | RIKSHIA | Body mass index based on weight and height at admission, mainly through asking patient (but could be measured) | Sensitivity: <18.4 \| 18.5-24.9 \| 25.0-29.9 \| 30.0+ \| NA | Main: Restricted cubic spline with five knots  Sensitivity: Categorial | Baseline |

## Supplementary Table 2 - Sensitivity analyses for intention-to-treat analysis with composite outcome

|  | **Beta blockers** | | **No beta blockers** | |  |
| --- | --- | --- | --- | --- | --- |
| **Sensitivity^a^** | **Events** | **Risk, %**  **(95% CI)** | **Events** | **Risk, %**  **(95% CI)** | **Hazard ratio   (95% CI)** |
| 1. Baseline covariates included in pooled logistic regression model | 637 | 10.0 (9.1, 11.0) | 88 | 11.6 (9.1, 14.5) | 0.81 (0.63, 1.11) |
| 1. a) Do not exclude individuals with a beta blocker before baseline (but adjust for prior beta blocker) | 1220 | 13.2 (12.4, 13.9) | 114 | 14.0 (10.8, 17.6) | 0.89 (0.68, 1.19) |
| b) Only exclude if beta blocker use on admission recorded in SWEDEHEART | 765 | 11.1 (10.2, 11.7) | 100 | 11.6 (8.9, 14.9) | 0.86 (0.65, 1.17) |
| 1. a) Complete case analysis | 364 | 9.5 (8.6, 10.6) | 57 | 11.3 (7.6, 15.2) | 0.75 (0.54, 1.09) |
| b) Categorization of continuous variables | 637 | 10.2 (9.5, 11.1) | 88 | 12.6 (9.1, 16.3) | 0.74 (0.56, 1.01) |
| 1. Identify day individuals become eligible, then clone and censor^b^ | 711 | 10.7 | 101 | 12.5 | 0.83 |

^a^ Confidence intervals estimated via bootstrapping with 200 samples ^b^ No confidence intervals estimated via bootstrapping due to computational restrictions

## Supplementary Table 3 - Sensitivity analyses for per-protocol analysis with composite outcome

|  | **Beta blockers** | | **No beta blockers** | |  |
| --- | --- | --- | --- | --- | --- |
| **Sensitivity^a^** | **Events** | **Risk, %**  **(95% CI)** | **Events** | **Risk, %**  **(95% CI)** | **Hazard ratio   (95% CI)** |
| Grace period of 90 days to define continuous treatment | 365 | 10.0 (8.9, 11.6) | 75 | 11.0 (7.5, 14.4) | 0.81 (0.61, 1.24) |

^a^ Confidence intervals calculated estimated via bootstrapping with 200 samples

## Supplementary Table 4 – ICD-10 and ATC codes used to define diagnoses and treatments for eligibility criteria and treatment strategies

| **Diagnoses** | **ICD-10 codes** |
| --- | --- |
| Bradycardia | R00.1, I49.5 |
| Av-block II-III | I44.1-3 |
| Hypotension | I95 |
| Syncope | R55.9, T67.1 |
| Asthma | J45-46 |
| Chronic Obstructive Pulmonary Disease | J44 |
| Stroke | I60-64 |
| Psychiatric Disorders | F01-F99 |
| Hypertension | I10, I15 |
| Angina | I20 |
| Arrhythmia | I47-49 |
| Heart Failure | I50 |
| **Treatments** | **ATC codes** |
| Metoprolol | C07AB02 |
| Bisoprolol | C07AB07 |
| Any beta blocker | C07 |
| Statins | C10AA |
| Antithrombotic treatments | B01 |

## Supplementary Table 5 - Baseline characteristics of eligible individuals for an emulation of a target trial of beta blockers vs. no beta blockers in Sweden, 2010-2017, continued from Table 2

|  | **Beta blockers** | **No beta blockers** | **Missing** | **SMD^a^** | **SMD after IP weighting^b^** |
| --- | --- | --- | --- | --- | --- |
|  | 9728 | 1198 |  |  |  |
| Hospital |  |  | 0 | 0.475 | 0.170 |
| Stockholm St Göran | 328 (3.4) | 37 (3.1) |  |  |  |
| Stockholm SÖS | 388 (4.0) | 44 (3.7) |  |  |  |
| Stockholm KI Solna | 416 (4.3) | 28 (2.3) |  |  |  |
| Stockholm KI Huddinge | 325 (3.3) | 21 (1.8) |  |  |  |
| Stockholm Danderyd | 533 (5.5) | 24 (2.0) |  |  |  |
| Uppsala | 319 (3.3) | 51 (4.3) |  |  |  |
| Eskiltuna | 302 (3.1) | 24 (2.0) |  |  |  |
| Linköping | 240 (2.5) | 41 (3.4) |  |  |  |
| Norrköping Vrinnevi | 85 (0.9) | 11 (0.9) |  |  |  |
| Motala | 42 (0.4) | 12 (1.0) |  |  |  |
| Jönköping | 132 (1.4) | 21 (1.8) |  |  |  |
| Eksjö | 48 (0.5) | 5 (0.4) |  |  |  |
| Ljungby | 36 (0.4) | 6 (0.5) |  |  |  |
| Kalmar | 291 (3.0) | 22 (1.8) |  |  |  |
| Kristianstad | 246 (2.5) | 23 (1.9) |  |  |  |
| Ängelholm | 75 (0.8) | 24 (2.0) |  |  |  |
| Malmö | 296 (3.0) | 39 (3.3) |  |  |  |
| Lund | 820 (8.4) | 121 (10.1) |  |  |  |
| Helsingborg | 246 (2.5) | 63 (5.3) |  |  |  |
| Halmstad | 248 (2.5) | 56 (4.7) |  |  |  |
| Varberg | 200 (2.1) | 31 (2.6) |  |  |  |
| Göteberg Sahlgrenska | 704 (7.2) | 60 (5.0) |  |  |  |
| Göteborg Östra | 234 (2.4) | 21 (1.8) |  |  |  |
| Göteborg Mölndal | 110 (1.1) | 17 (1.4) |  |  |  |
| Kungälv | 149 (1.5) | 15 (1.3) |  |  |  |
| Borås | 112 (1.2) | 9 (0.8) |  |  |  |
| Alingsås | 91 (0.9) | 16 (1.3) |  |  |  |
| Lidköping | 113 (1.2) | 24 (2.0) |  |  |  |
| Skövde | 347 (3.6) | 41 (3.4) |  |  |  |
| Karlstad | 433 (4.5) | 68 (5.7) |  |  |  |
| Örebro | 252 (2.6) | 25 (2.1) |  |  |  |
| Västerås | 236 (2.4) | 27 (2.3) |  |  |  |
| Köping | 90 (0.9) | 9 (0.8) |  |  |  |
| Falun | 325 (3.3) | 64 (5.3) |  |  |  |
| Mora | 80 (0.8) | 13 (1.1) |  |  |  |
| Sundsvall | 264 (2.7) | 9 (0.8) |  |  |  |
| Östersund | 137 (1.4) | 21 (1.8) |  |  |  |
| Umeå | 191 (2.0) | 4 (0.3) |  |  |  |
| Sunderbyn | 244 (2.5) | 51 (4.3) |  |  |  |
| Year of index |  |  | 0 | 0.197 | 0.089 |
| 2010 | 408 (4.2) | 32 (2.7) |  |  |  |
| 2011 | 1304 (13.4) | 143 (11.9) |  |  |  |
| 2012 | 1393 (14.3) | 137 (11.4) |  |  |  |
| 2013 | 1326 (13.6) | 144 (12.0) |  |  |  |
| 2014 | 1391 (14.3) | 177 (14.8) |  |  |  |
| 2015 | 1438 (14.8) | 186 (15.5) |  |  |  |
| 2016 | 1450 (14.9) | 192 (16.0) |  |  |  |
| 2017 | 1018 (10.5) | 187 (15.6) |  |  |  |
| ^a^SMD - Standardized mean difference | | | |  |  |
| ^b^IP - Inverse probability | | | |  |  |

##
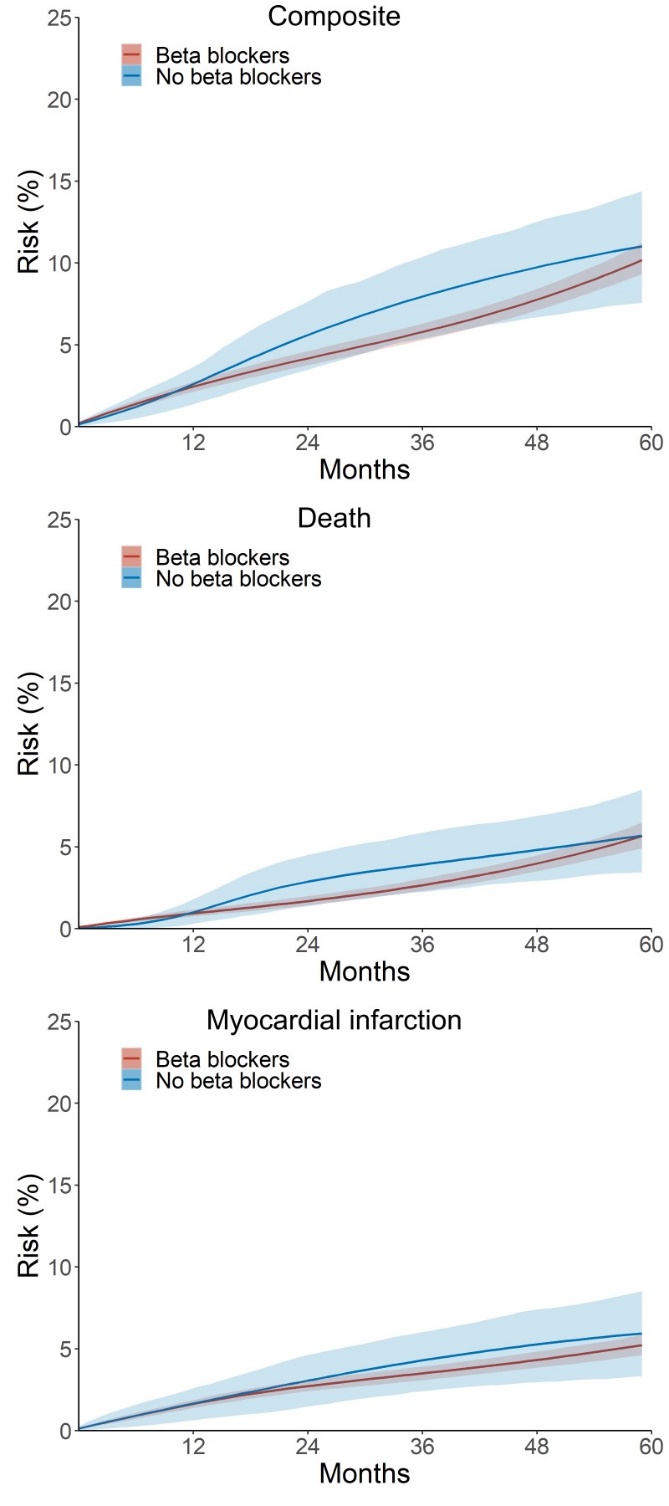
Supplementary Figure 1 - Estimated risk of death and myocardial infarction under beta blockers versus no beta blockers. Per protocol analysis of an emulated target trial in individuals with myocardial infarction with preserved ejection fraction in Sweden, 2011–2017 (shaded intervals represent limits of the pointwise 95% CIs)

|  |
| --- |
